# Concurrent image-guided intensity modulated radiotherapy and chemotherapy following neoadjuvant chemotherapy for locally advanced nasopharyngeal carcinoma

**DOI:** 10.1186/1748-717X-6-95

**Published:** 2011-08-13

**Authors:** Pei-Wei Shueng, Bing-Jie Shen, Le-Jung Wu, Li-Jen Liao, Chi-Huang Hsiao, Yu-Chin Lin, Po-Wen Cheng, Wu-Chia Lo, Yee-Min Jen, Chen-Hsi Hsieh

**Affiliations:** 1 Division of Radiation Oncology, Department of Radiology, Far Eastern Memorial Hospital, Taipei, Taiwan; 2 Department of Otolaryngology, Far Eastern Memorial Hospital, Taipei, Taiwan; 3 Division of Medical Oncology and Hematology, Department of Internal Medicine, Far Eastern Memorial Hospital, Taipei, Taiwan; 4 Departments of Radiation Oncology, Tri-Service General Hospital, National Defense Medical Center, Taipei, Taiwan; 5 Institute of Traditional Medicine, School of Medicine, National Yang-Ming University, Taipei, Taiwan

**Keywords:** Concurrent chemoradiation, Intensity-modulated radiotherapy, Helical tomotherapy, Nasopharyngeal carcinoma

## Abstract

**Background:**

To evaluate the experience of induction chemotherapy followed by concurrent chemoradiationwith helical tomotherapy (HT) for nasopharyngeal carcinoma (NPC).

**Methods:**

Between August 2006 and December 2009, 28 patients with pathological proven nonmetastatic NPC were enrolled. All patients were staged as IIB-IVB. Patients were first treated with 2 to 3 cycles of induction chemotherapy with EP-HDFL (Epirubicin, Cisplatin, 5-FU, and Leucovorin). After induction chemotherapy, weekly based PFL was administered concurrent with HT. Radiation consisted of 70 Gy to the planning target volumes of the primary tumor plus any positive nodal disease using 2 Gy per fraction.

**Results:**

After completion of induction chemotherapy, the response rates for primary and nodal disease were 96.4% and 80.8%, respectively. With a median follow-up after 33 months (Range, 13-53 months), there have been 2 primary and 1 nodal relapse after completion of radiotherapy. The estimated 3-year progression-free rates for local, regional, locoregional and distant metastasis survival rate were 92.4%, 95.7%, 88.4%, and 78.0%, respectively. The estimated 3-year overall survival was 83.5%. Acute grade 3, 4 toxicities for xerostomia and dermatitis were only 3.6% and 10.7%, respectively.

**Conclusion:**

HT for locoregionally advanced NPC is feasible and effective in regard to locoregional control with high compliance, even after neoadjuvant chemotherapy. None of out-field or marginal failure noted in the current study confirms the potential benefits of treating NPC patients by image-guided radiation modality. A long-term follow-up study is needed to confirm these preliminary findings.

## Background

Locally advanced NPC patients present with poor prognosis. This has led to increasing interest in exploring the use of chemotherapy. Recently, meta-analysis has confirmed the superiority of concurrent chemoradiation (CCRT) over radiotherapy (RT) alone in terms of survival or locoregional control among patients with locally advanced NPC [1-3]. However, the optimal regimen and scheduling remains to be determined and efforts to improve the increased toxicities are still unremitting.

With the improvement of RT techniques, such as intensity-modulated radiotherapy (IMRT) or image-guided radiotherapy (IGRT), radiation oncologists have the ability to deliver tumoricidal doses to the target while maintaining tolerable doses to critical organs. Recently, several non-randomized studies have demonstrated impressive tumor control and survival using IMRT in NPC. Moreover, the predominant failure pattern is now distant failure rather than local failure [4]. To conquer distant metastasis, adding induction chemotherapy or adjuvant chemotherapy to concurrent chemoradiation is still an attractive approach that needs to be clarified.

Helical tomotherapy (HT), an innovative image-guided IMRT device, can perform daily CT image registration before treatment and deliver 51-angled rotational IMRT. Our institute started the first HT treatment using Tomotherapy Hi-Art systems (Tomotherapy, Madison, WI) in December 2006. Using HT, we have previously reported encouraging experiences for oropharyngeal [5], postoperative treatment of high-risk oral cavity cancer [6] and cervical cancer [7]. In comparison with conventional IMRT, the HT results have demonstrated better dosimetry coverage and highly conformal dose distributions to the targets and the impressive ability to simultaneously spare critical organs. In the treatment of nasopharyngeal carcinoma, tomotherapy plans were superior to IMRT plans in conformity and homogeneity of planning target volume (PTV) and the sparing of the critical organs at risk (OARs) [8].

We herein report our preliminary experience of concurrent helical tomotherapy plus chemotherapy following induction chemotherapy for locally advanced NPC, with special focus on response rate, acute treatment-related sequelae and failure pattern and locoregional control.

## Methods

### Patient Characteristics

Between August 2006 and December 2009, 28 patients with pathological proven NPC were enrolled in this retrospective analysis. All of the patients were diagnosed as non-metastatic NPC in the cancer work-up initially. Approval for the study was obtained from the Institutional Review Board of Far Eastern Memorial Hospital (FEMH No. 100050-E). The clinical characteristics are detailed in Table 1. There were 22 men and 6 women with a median age of 47.5 years. Most patients (85.7%) had pathology of WHO type III (undifferentiated carcinoma). Patients were staged according to the 2002 American Joint Committee on Cancer (AJCC) staging system. All patients were staged as having locally advanced disease (stage IIB-IVB). Table 2 detailed the TNM distribution of the patients.

**Table 1:** Characteristics of 28 patients

Variable	number	percent
Gender		
Male	22	78.6%
Female	6	21.4%
Stage (AJCC, 2002)		
IIB	3	10.7%
III	15	53.6%
IVA/B	10	35.7%
T stage		
T1-T2	11	39.3%
T3-T4	17	60.7%
N stage*		
N0-N1	8	28.6%
N2-N3	20	71.4%
Field-dose arrangement		
SIB	24	85.7%
Conventional shrinking field	4	14.3%
Pathology		
WHO I & II	4	14.3%
WHO III	24	85.7%

**Table 2:** Dose-volumetric statistics for target volumes

Parameters	Mean (range)	
	
	PTV_70_	PTV_63_
Volume (cc)	253.8 (61.7-776.1)	528.8 (175.8-1213.2)
Mean dose (Gy)	71.9 (70.1-75.3)	64.3 (54.2-68.8)
Maximum dose (Gy)	74.4 (70.3-79.7)	69.4 (54.6-76.1)
Minimum dose (Gy)	60.1 (44.9-69.7)	47.4 (26.8-57.6)
D_95 _(Gy)	70.1 (68.8-72.0)	61.4 (53.9-67.0)
V_97 _(%)	98.3 (95.3-100.0)	97.8 (94.6-100.0)

Staging workups included complete histories and physical examinations, fiberoptic endoscopic evaluation, complete blood counts, liver and renal function tests, chest X-rays, abdominal ultrasound, magnetic resonance imaging (MRI) scans of the head and neck region, bone scan and dental evaluation. CT scans of the chest and abdomen were obtained whenever possible before the beginning of treatment if distant metastasis was suspected by abnormal finding in chest x-ray or abdominal ultrasound.

### Chemotherapy

All patients were treated with induction chemotherapy followed by CCRT with HT. Induction chemotherapy regimens, EP-HDFL, consisted of Epirubicin 40 mg/m^2^, 30 minutes infusion, followed by Cisplatin 60 mg/m^2^, 5-FU 2000 mg/m^2^, and Leucovorin 300 mg/m^2^, 24 hours infusion on day 1, and 5-FU 2000 mg/m^2^, and Leucovorin 300 mg/m^2^, 24 hours infusion on day 8 and 15, repeated every 4 weeks. Three cycles were planned unless severe side effects occurred. Chemotherapy during the CCRT phase, PFL, consisted of Cisplatin 30 mg/m^2^, 5-FU 450 mg/m^2 ^as bolus, and Leucovorin 30 mg/m^2^, on a weekly basis. Curative radiotherapy began within 3 weeks after completion of the last cycle of induction chemotherapy.

### Radiotherapy

#### Immobilization and Contouring

Patients were immobilized using perforated Type-S thermoplastic head frames (MT-CFHN-C; Civco Medical Solutions, Kalona, IA) for head and shoulder immobilization after induction chemotherapy completed. The head frames would be corrected after a significant neck burden reduction during CCRT. A volumetric contrast enhanced CT image in serial 3 mm slices was acquired for treatment planning.

#### Target and Normal Tissue Volume Delineation and Constraints

Target objects and normal structures were outlined slice by slice on the treatment planning CT. On several occasions, RT-planning images were fused with diagnostic MRI to improve target delineation.

The gross tumor volume (GTV) encompassed the gross extent of the primary tumor and involved neck nodes shown by imaging before induction chemotherapy as well as physical examination. Whenever possible, MRI scan done before induction chemotherapy (24/28) was used in addition to the CT scan to delineate the GTV with the assistance of a neuroradiologist. A GTV node was outlined to have a nodal size larger than 10 mm in the short-axis diameter or the presence of central lucency on CT or MRI images. The clinical target volume of 70 Gy (CTV70) included the GTV with an additional 10 mm margin and GTV of node with an expansion of 5 mm, respectively. The clinical target volume of 63 Gy (CTV63) was designed to include areas at risk for microscopic involvement, as well as the entire nasopharynx, retropharyngeal nodal regions, skull base, clivus, pterygoid fossae, parapharyngeal space, sphenoid sinus, the posterior one third of the nasal cavity/maxillary sinuses that includes the pterygopalatine fossae, and levels I through V nodal regions. Level II nodes were contoured bilaterally to the base of skull. The clinical target volume of 56 Gy (CTV56) was designed for the low-risk subclinical disease area. To account for organ motion and patient setup errors, all of the PTV70, PTV63 and PTV56 were defined as CTV plus a margin of 3 mm. For patients treated with the whole-field SIB technique, PTV70, PTV63 and PTV56 were delivered in the same days and all were amenable to be completed in 35 fractions within 7 weeks.

Critical structures included the brainstem, spinal cord, brain, lens, eyeballs, optic chiasma, optic nerve, inner ear, oral cavity, mandible, parotid gland, larynx, and lung. Optimization was performed using the following criteria for dose constraints. The dose constraints for OARs were as follows: (1) brainstem: maximum dose 50 Gy, (2) spinal cord: maximum dose 40 Gy, (3) optic chiasm and optic nerve: maximum dose 45 Gy, (4) mandible: maximum dose 70 Gy or 1 cm^3 ^or less for 70 Gy or more, (5) bilateral parotid glands: mean dose less than 30 Gy, and median dose less than 26 Gy, and whole parotid gland volume, with a dose less than 20 Gy, more than 20 cm^3^, and (6) middle and inner ear: mean dose less than 50 Gy. The planning OAR volume (PRV) was set as the brain stem and spinal cord with 5-mm margins in the axial plane. The PRVs of the chiasma and optic nerve were set with 3-mm margins in every direction.

#### Treatment Plan and Delivery

The field width, pitch, and modulation factor usually used for treatment planning optimization were 2.5 cm, 0.32, and 3.0, respectively. Maximum importance was given to target dose coverage. The constraints on dose and penalty were adjusted accordingly during optimization. All patients received daily megavoltage CT acquisitions for setup verification.

### Follow-up

The response criteria were as follows: a complete response was defined as complete regression of all evidence of disease; a partial response required a 50% decrease of the summed products of the two largest perpendicular diameters of all measurable lesions, without an increase in size of more than 25% in any lesion or the appearance of new lesions; stable disease was defined as no significant change or any change in tumor size that was less than a partial response but not large enough to be considered progressive disease; and progressive disease was defined as an increase of at least 25% in the size of measurable lesions or the appearance of any new lesion. Response was assessed before the initiation of radiotherapy and 3 months after completion of the treatment, respectively.

The acute toxicity occurring within 90 days since the beginning of RT was assessed weekly throughout the treatment. The toxicities were defined and graded according to the Common Terminology Criteria for Adverse Events, version 3.0 [9].

### Statistical methods

Descriptive statistics (mean, median, and proportions) were calculated to characterize the patient, disease, and treatment features, as well as toxicities after treatment. The OS, PFS, LRPF, and DMF rates were estimated using the Kaplan-Meier product-limit method [10]. Freedom from local progression was defined as the absence of primary tumor upon physical examination and radiographic examination (CT and MRI scan). Durations were calculated from the date of pathologic proof. Differences were considered significant at p < 0.05. MedCalc statistical software (version 11.2.1.0, MedCalc Software, Mariakerke, Belgium) was used for conducting statistical analyses, manipulating data, and generating tables and graphs that summarize data.

## Results

### Dose-volume analysis

Dose-volume histograms statistics for the PTV and organs at risk (OARs) are described in table 2 and 3, respectively. The D95 in PTV70 ranged from 68.8 Gy (98% of the prescription dose) to 72 Gy (100% of the prescription dose). The V97 in PTV70 ranged from 97.3% to 100%. Mean doses to parotid glands were 33.7 Gy (25.90-43.49 Gy) for the right and 34.1 Gy (24.02-48.72 Gy) for the left. The other OARs are summarized in Table 3.

**Table 3:** Dose-volumetric statistics for organs at risk (OARs)

Organs	Mean (range)
Spinal cord [D_max_(Gy)]	40.70 (29.20-53.84)
Brainstem [D_max_(Gy)]	50.15 (31.49-62.01)
Right Optic nerve [D_max_(Gy)]	46.08 (19.40-76.39)
Left Optic nerve [D_max_(Gy)]	43.75 (7.98-72.70)
Optic chiasm [D_max_(Gy)]	46.49 (25.50-73.06)
Right inner ear	
D_max _(Gy)	59.40 (47.61-73.53)
D_mean _(Gy)	41.87 (24.43-65.37)
Left inner ear	
D_max _(Gy)	60.26 (40.87-74.55)
D_mean _(Gy)	43.26 (23.01-70.41)
Right parotid gland	
D_mean _(Gy)	33.71 (25.90-43.49)
V_30 Gy _(%)	45.64 (29.30-60.00)
Left parotid gland	
D_mean _(Gy)	34.09 (24.02-48.72)
V_30 Gy _(%)	46.38 (27.10-78.39)

### Response

Most patients (85.7%) were treated with Whole-field SIB (simultaneous-integrated boost) HT techniques. The median follow-up duration was 33 months (range: 13 to 53 months). Thirteen patients received 2 cycles of induction chemotherapy due to severe nausea (1/28), neutropenia (1/28), sepsis (1/28) and partial response with unsatisfactory response judged by medical oncologist (10/28). The remaining underwent 3 cycles of chemotherapy. Primary tumors had a higher response rate to induction chemotherapy (96.4%) compared with nodal disease (80.8%) which was evaluated by endoscopy & CT or MR for all patients. The complete response rates were 39% and 27% for the primary tumor and neck node, respectively. (Table 4) No patients experienced disease progression during chemotherapy. Also, after remission via induction chemotherapy, there were no patients who had primary or lymph node enlargement during the rest period before CCRT.

**Table 4:** Clinical response after induction chemotherapy and 2 months after completion of CCRT

Response	After Induction	After Concomitant
No. (%)	Chemoradiation	chemotherapy
Nasopharynx, by endoscopy & CT or MR		
SD	1/28(3.6%)	0/28(0.0%)
PR	16/28(57.1%)	2/28(7.1%)
CR	11/28(39.3%)	26/28(92.9%)
Neck node, by CT or MR		
SD	5/26*(19.2%)	0/26(0.0%)
PR	14/26(53.8%)	5/26(19.2%)
CR	7/26(26.9%)	21/26(80.8%)

*Two patients were staged as T4N0, so 26 patients were available for nodal evaluation.

Abbreviations:

SD: stable disease, PR: partial response, CR: complete response.

After induction chemotherapy, all patients also received CCRT with HT and achieved complete or partial remission either in the primary site or gross neck nodes. The median cycles for patients received chemotherapy during RT were 4 cycles (range: 2-7 cycles). There were 4 (14.3%), 2 (7.1%) and 3 (10.7%) of patients received chemotherapy during RT with 5, 6 and 7 cycles, respectively. The average weeks for CCRT were 7.8 ± 1.1 wks (range: 6-10 wks). There were 7 (23.3%) and 2 (6.7%) patients completed the CCRT course within 9 and 10 wks, respectively. The complete response rate of the nodal area (80.8%) was inferior to primary location (92.9%). After completion of the whole treatment, small residual tumors were noted either at the primary site or neck with 7.1% and 19.2% of patients, respectively. These residual tumors all showed complete regression upon follow-up after 3 months (Table 4).

### Treatment outcome

**Figure 1:**
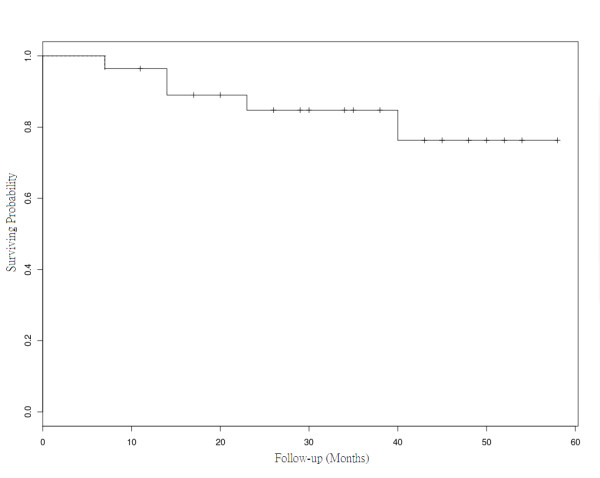
**The actuarial overall survival rates at 3 years**.

The estimated 3-year progression-free (PF) rates for local, regional, locoregional and DMF survival rate were 92.4%, 95.7%, 88.4%, and 78.0%, respectively. The 3-year estimates of locoregional PF for patients with stage II-IV disease were 100%, 92.9%, and 76.2%, respectively. The 3- year estimated OS was 83.5% (Figure 1). No patient was lost as of follow-up. Three patients and 2 patients died by distant failure and intercurrent disease (one of chemotherapy related septic shock and the other died of cardiac dysfunction probably related to the anthracycline-chemotherapy of cardiac [11]), respectively.

### Acute Toxicities

The median treatment period during CCRT was 54 days (range: 42 to 73 days). No fatal toxicity related to the planned treatment occurred in this study. Before induction chemotherapy, all but 3 patients had normal hemogram (Table 5). During induction chemotherapy, grade 3 leukopenia occurred in 1 patient. No patients experienced grade 3 anemia or grade 3 thrombocytopenia. However, the low toxicities of induction chemotherapy are probably due to the low doses of CDDP and Epirubicin.

**Table 5:** Acute hematological toxicities in 28 patient after induction chemotherapy and concurrent chemoradiation according to CTCAE v3.0

Item	anemia			leucopenia			thrombocytopenia		
			
Interval	before	IC	CCRT	before	IC	CCRT	before	IC	CCRT
Grade									
0	25(89.3%)	11(39.3%)	7(25.0%)	28(100.0%)	19(67.9%)	8(29.6%)	27(96.4%)	17(60.7%)	13(46.4%)
1	1(3.6%)	13(46.4%)	10(35.7%)	0	5(17.9%)	4(14.8%)	1(3.6%)	11(39.3%)	10(35.7%)
2	1(3.6%)	4(14.3%)	10(35.7%)	0	3(10.7%)	12(44.4%)	0	0	5(17.9%)
3	0	0	1(3.6%)	0	1(3.6%)	4(11.1%)	0	0	0
4	1(3.6%)	0	0	0	0	0	0	0	0

Abbreviations:

CTCAE v3.0: the Common Terminology Criteria for Adverse Events, version 3.0. IC: induction chemotherapy (IC); CCRT: concurrent chemoradiation.

For CCRT with HT, 4 patients (14.3%) developed grade 3 leukopenia and 1 patient (3.6%) developed grade 3 anemia during treatment. Acute nonhematological toxicities related to radiotherapy, including xerostomia and dermatitis, were mostly mild (Table 6.) Only 1 patient had grade 3 xerostomia. Grade 3 or 4 dermatitis was noted in 2 and 1 patients, respectively. However, 13 patients (46.4%) suffered from grade 3 mucositis. Other grade 3 reactions such as dysphagia and weight loss were noted in 4 and 2 patients, respectively. Seven patients (25.0%) needed NG feeding for nutritional supports.

**Table 6:** Acute radiation-related toxicities according to CTCAE v3.0

	Acute toxicities				
	
Grade	xerostomia	mucositis	dysphagia	dermatitis	weight loss
0	0	0	1(3.60%)	0	2 (7.1%)
1	13(46.4%)	4(14.3%)	8(28.6%)	17(60.7%)	12(42.9%)
2	14(50.0%)	11(39.8%)	15(53.6%)	8(28.6%)	12(42.9%)
3	1(3.6%)	13(46.4%)	4(14.3%)	2(7.1%)	2(7.1%)
4	0	0	0	1(3.6%)	0

Abbreviations:

CTCAE v3.0: the Common Terminology Criteria for Adverse Events, version 3.0.

### Late Toxicities

For CCRT with HT, none of patients developed grade 3 toxicities related to radiotherapy, including xerostomia, dysphagea, dry eyes, trismus and hearing loss. Most of them are normal to grade 1 of toxicities. Only 4/28 patient had grade 2 xerostomia and 1/28 had grade 2 hearing loss.

### Failure pattern

There were 89.3% (25/28) without locoregional failure. The failure pattern disclosed as follows: local failure only, 2 patients (7.1%); regional failure only, 1 patient (3.6%); distant metastases only, 4 patients (14.3%); and no local plus regional and/or distant failure.

**Figure 2:**
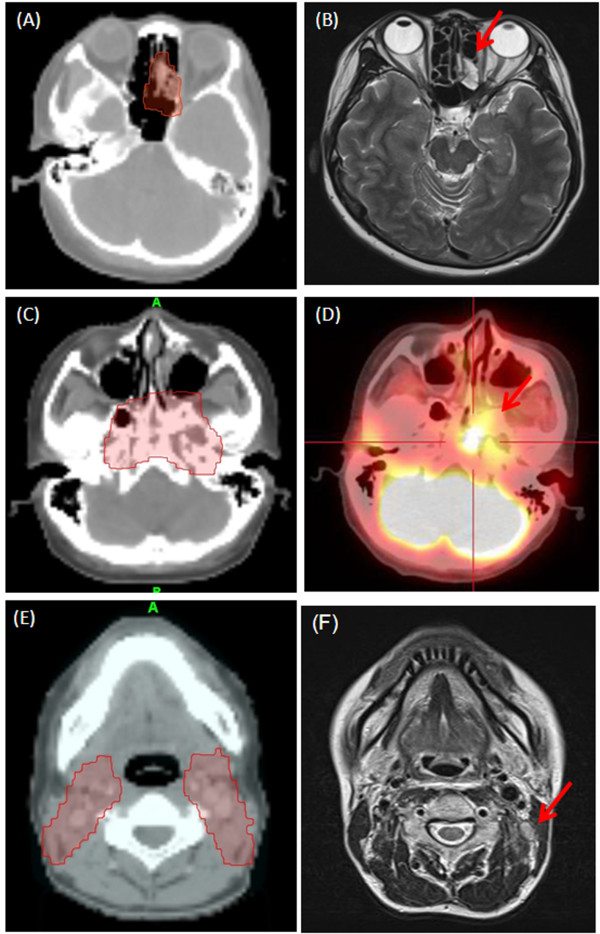
**The comparison of original planning dose distribution (red color area) and locoregional failure (red arrow)**. For patient 1. (A) In 2007/4, planning Dose distribution of 70 Gy (red color area) at ethmoid sinus; (B) In 2008/1, MRI images show a local relapse. For patient 2. (C) In 2008/11, planning dose distributions of 71.6 Gy (red color area) at skull base; (D) In 2009/3, PET-CT images show a at skull base, SUV_max _= 6.4. For patient 3. (E) In 2008/7, planning Dose distributions of 70 Gy (red color area) at neck lymph node and lymphatic drainage; (F) In 2009/6, MRI images show a regional lymph node relapse.

One patient with initial stage IV disease (cT4N1M0) failed locally at the ethmoid sinus 10 months post treatment. After functional endoscopic sinus surgery and adjuvant chemotherapy, the disease was well controlled. (Figure 2A and 2B) Another patient with stage IV disease (cT4N3bM0) failed at the nasopharynx 14 months after the treatment. She then underwent local irradiation plus cetuximab and chemotherapy but died of septic shock. (Figure 2C and 2D)

The only patient who failed for nodal disease with initial stage III disease (cT3N2M0) suffered from left upper neck relapse 16 months after completion of treatment and then was successfully salvaged by neck dissection. (Figure 2E and 2F) No adjuvant treatment was done since only one of 16 dissected nodes showed metastatic lesion. No extracapsular extension or other pathological risk factors were noted. The failure was in a previously irradiated field. No patients failed at the field margins or out of RT fields. The local and regional controls after salvage treatment were 96.4% and 100%, respectively.

Four patients developed distant metastases over the bone, liver, liver plus bone, and lung at the 6th, 7th, 10th and 40th month after completion of treatment. All of these 4 patients had N2 disease (stage III and IV). The average relapse time was 8 months. Three of them were died of disease progression and only one patient with liver metastases is still alive with disease and now under systemic treatment. We observed no parotid or dermal failure.

## Discussion

Impressive clinical data of NPC treated by IMRT have been reported in recent years. In one study, the 4-year local progression-free and regional progression-free rates for loco-regional advanced NPC patients were 97% and 98%, respectively [12]. Recent results from Hong Kong and the Memorial Sloan-Kettering cancer center have also shown similar findings [13-15]. However, with integration of aggressive concurrent chemoradiotherapy schedules, the changing failure pattern has been noted in several publications [12,16,17] and the distant metastases rates, nevertheless, can be as high as 30% [4].

To conquer the problem of distant metastases, adding neoadjuvant chemotherapy or adjuvant chemotherapy with concurrent chemoradiation is still an attractive approach that needs to be clarified, although post experience is very sparse. A study conducted in Hong Kong [18] reported that 24/25 locally advanced NPC patients achieved partial remissions after induction chemotherapy. Additionally, the 3-year local-PF, regional-PF, and DM-PF survival rates were 89.6%, 87.2%, and 80.4%, respectively. China has report the largest series of concurrent chemotherapy and IMRT data, with 323 locoregionally advanced NPC patients with neoadjuvant or adjuvant chemotherapy [19]. The overall 3-year local-PF, regional-PF, DM-PF, and overall survival rates were 93.6%, 93.3%, 86.6%, and 87.2%, respectively. A study in Japan demonstrated the first experience of HT plus chemotherapy for 20 patients with a limited observation period. However, 18 patients who underwent chemotherapy with NDP (*cis*-diammineglycolatoplatinum, Nedaplatin) and 5FU were in alternating settings. During the alternating chemoradiotherapy and with a median FU of 10.9 months, one patient failed in the regional node and another one failed in the liver. The 10-month OS was 95% [20]. In the current study, induction chemotherapy and CCRT with HT were well tolerated. During neoadjuvant chemotherapy, only one patient occurred grade 3 leukopenia. No patients experienced grade 3 anemia or thrombocytopenia. Four patients developed grade 3 leukopenia and 1 patient developed grade 3 anemia during the following CCRT with HT treatment. The median treatment time for CCRT was 54 days. The estimated 3-year PF for local, regional, and locoregional survival rates were 92.4%, 95.7%, and 88.4%, respectively. HT for locoregionally advanced NPC was shown to be feasible and effective in regard to locoregional control with high compliance, even after neoadjuvant chemotherapy.

Even though nearly 90% of our patients had locally advanced disease (stage III and IV), patients had excellent locoregional control rates after HT plus chemotherapy or even salvage therapy. However, of the 7 relapsed patients in the current study, 4 patients presented distant metastases. The regiment of induction chemotherapy in the current study was CDDP/Epirubicin/5-FU/Leucovoren (60/40/2000/300 mg/m2). Compared to the other studies, the doses of CDDP and Epirubicin in the current study were lower than the other studies [14,21,22]. The 3-year DMF survival rate was 78%, suggesting that distant metastases are still the major obstacle to be broken through. Moreover, present regimens of chemotherapy are not effective enough in preventing distant metastases and should be reevaluated.

Higher irradiation doses deliver high rates of locoregional control, progression-free survival for head and neck cancer [23]. However, we may need to be concerned about late complications if the dose is escalated and the volume of tissues are exposed to high doses [24]. On the other hand, if the volume of tissues exposed to high doses is reduced with image-guided IMRT, there is a possibility that treatment could achieve higher locoregional control rate and the probability of such complications could be reduced simultaneously. In the current study, the locoregional failure of 3 patients all belonged to in-field failure. The D95 in PTV70 ranged from 68.8 Gy to 72 Gy and the V97 in PTV70 ranged from 97.3% to 100%, respectively. (Table 2) None of the out-field or marginal failures noted in the current study showed 3 mm of PTV's margin, confirming the potential benefits of treating NPC patients with image-guided radiation modality. This finding also suggests that using 3 mm as the PTV margin in image-guided radiation therapy settings is feasible. Additionally, limited grade 3 of xerostomia, dysphagia and dermatitis were noted in the current setting (Table 6). Moreover, most of patients are normal to grade 1 of late toxicities. Only 4/28 patient had grade 2 xerostomia and 1/28 had grade 2 hearing loss. With accurate image-guided modality, dose escalation with reduced increase of toxicity for OARs becomes more reliable, providing relief for locoregionally advanced NPC patients.

One patient died of cardiac dysfunction, and the possibility that the toxicity was related to epirubicin used in induction chemotherapy should be considered. The range of total dose for epirubicin that cause cardiac toxicities is around 560-600 mg/m^2^[11,25]. Bonneterre J, et al [25] reported that there were 2/85 cases of congestive heart failure observed after adjuvant treatment with six cycles of fluorouracil 500 mg/m^2^, epirubicin 100 mg/m^2^, and cyclophosphamide 500 mg/m^2 ^for breast cancer patients in the 8 years follow up. Hasbini A, et al [11] used mitomycin, 5-fluorouracil, epirubicin, and cisplatin to treat recurrent and metastatic undifferentiated carcinoma of nasopharyngeal and one 42-year-old patient died of cardiac failure which was probably related to the anthracycline-chemotherapy. In the current study, three cycles of 40 mg/m^2 ^induction epirubicin was prescribed and the total dose was 120 mg/m^2^. Although, the total dose of epirubicin is far from the doses that cause cardiac toxicity.

Even though this innovative approach acquired favorable outcomes with impressive locoregional control and survival result, several limitations need to be addressed. First, our study was retrospective and was carried with inherent biases usual to such a study design. Second, our sample size was small. Thus, these findings should be considered as preliminary and in need of validation in a larger patient group. Third, the study lacked in-house comparable results such as tomotherapy versus conventional IMRT or current regimen versus concurrent chemoradiation. Furthermore, the observation of long-term toxicities should be reported in the future. The clinical benefit of modern IGRT using tomotherapy, hence, could not be fully determined. Due to these limitations, this combination protocol must not be used in the daily practice of treatment for locally advanced NPC.

## Conclusions

In conclusion, this is the first report providing evidence that HT for locoregionally advanced NPC is feasible and effective in regard to locoregional control with high compliance, even after neoadjuvant chemotherapy. No out-field or marginal failure was noted in the current study, confirming the potential benefits of treating NPC patients with image-guided radiation modality. A long-term follow-up study is needed to confirm these preliminary findings.

## Competing interests

We have no personal or financial conflict of interest and have not entered into any agreement that could interfere with our access to the data in the research, or upon our ability to analyze the data independently, to prepare manuscripts, and to publish them.

## Authors' contributions

PWS and BJS drafted the manuscript. LJW, CHH, LJL, PWC, WCL, YMJ and YCL participated in taking care of patients. CHH and PWS carried out all CT evaluations, study design, target delineations and interpretation of the study. CHH conceived of the study, and participated in its design and coordination. All authors read and approved the final manuscript.
